# Do-not-attempt-resuscitation decision making: physicians’ recommendations differ from the GO-FAR score predictions

**DOI:** 10.1186/s12245-024-00669-3

**Published:** 2024-07-11

**Authors:** David Olukolade Alao, Snaha Abraham, Emad Dababneh, Roxanne Roby, Mohammed Farid, Nada Mohammed, Natalia Rojas-Perilla, Arif Alper Cevik

**Affiliations:** 1 Tawam Hospital, Al Ain, UAE; 2 Department of Internal Medicine, Section of Emergency Medicine, College of Medicine and Health Sciences, United Arab Emirates University, Al Ain, UAE; 3 Statistics Support Center, United Arab Emirates University, Al Ain, UAE; 4 Life Support Center, Tawam Hospital, Al Ain, UAE

**Keywords:** DNR, IHCA, GO-FAR score, Physician decision-making

## Abstract

**Background and aim:**

In-hospital cardiac arrest (IHCA) is a major cause of mortality globally, and over 50% of the survivors will require institutional care as a result of poor neurological outcome. It is important that physicians discuss the likely outcome of resuscitation with patients and families during end-of-life discussions to help them with decisions about cardiopulmonary resuscitation. We aim to compare three consultants’ do-not-resuscitate (DNR) decisions with the GO-FAR score predictions of the probability of survival with good neurological outcomes following in-hospital cardiac arrest (IHCA).

**Methods:**

This is a retrospective study of all patients 18 years or older placed on a DNR order by a consensus of three consultants in a tertiary institution in the United Arab Emirates over 12 months. Patients’ socio-demographics and the GO-FAR variables were abstracted from the electronic medical records. We applied the GO-FAR score and the probability of survival with good neurological outcomes for each patient.

**Results:**

A total of 788 patients received a DNR order, with a median age of 71 years and a majority being males and expatriates. The GO-FAR model categorized 441 (56%) of the patients as having a low or very low probability of survival and 347 (44%) as average or above. There were 219 patients with a primary diagnosis of cancer, of whom 148 (67.6%) were in the average and above-average probability groups. There were more In-hospital deaths among patients in the average and above-average probability of survival group compared with those with very low and low probability (243 (70%) versus 249 (56.5%) (*P* < 0.0001)). The DNR patients with an average or above average chance of survival by GO-FAR score were more likely to be expatriates, oncology patients, and did not have sepsis.

**Conclusions:**

The GO-FAR score provides a guide for joint decision-making on the possible outcomes of CPR in the event of IHCA. The physicians’ recommendation and the ultimate patient’s resuscitation choice may differ due to more complex contextual medico-social factors.

## Introduction

In-hospital cardiac arrest (IHCA) is a major cause of mortality globally, with an estimate of over 292,000 IHCA occurring in the United States annually [1]. Over 50% of the survivors have poor functional outcomes, with the required institutional care placing a significant burden on the health economy [2]. The economic cost of cardiac arrest includes the cost of hospital admission and post-discharge costs of hospital visits, health visits and welfare dependency. A recent Danish study estimated the mean cost of OHCA survivors over six years to be over 119,000 Euros. [3]

Effective Cardio-pulmonary resuscitation (CPR) is the cornerstone of the management of IHCA.

Following decades of the practice of CPR and improvement in resuscitation medicine, the reported survival rates for In-Hospital Cardiac Arrest remains poor [4–7]. A more rational application of CPR became necessary following the recognition of patients’ rights to determine care preferences and enacting laws allowing doctors to determine care’s futility. It is important to ensure that patients with a poor chance of survival or poor neurological outcomes are not subjected to futile CPR. As a result, many countries worldwide now have laws supporting do-not-resuscitate (DNR) when there is a patient’s wish against CPR or when there are clear indications of poor patient outcomes [8, 9].

However, many developing countries do not have DNR laws and statutes [4, 10].

**Fig. 1:**
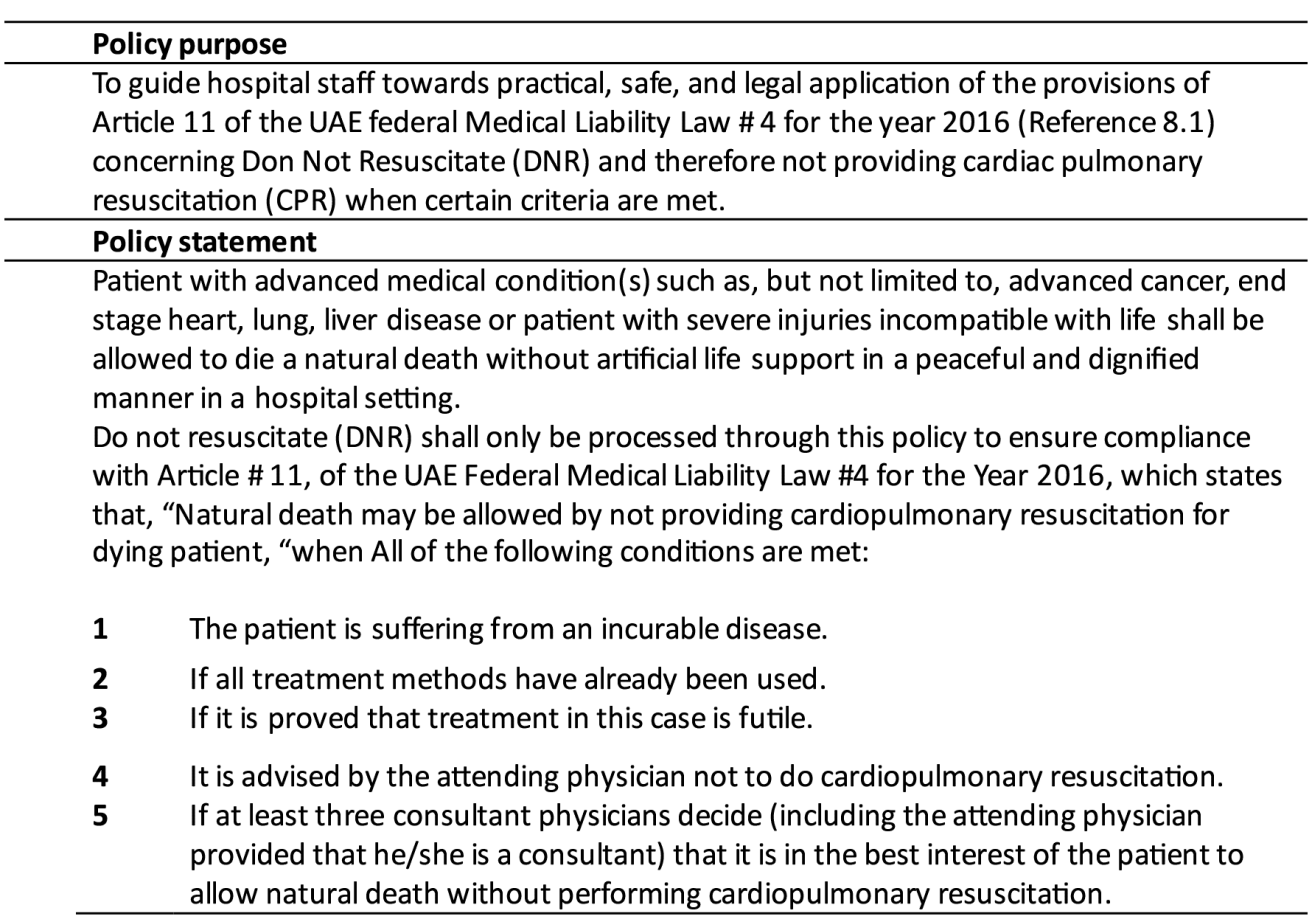
DNR law in the United Arab Emirates

That was the case in the United Arab Emirates (UAE) before the DNR law was enacted in April 2020 [Fig. 1]. The law stipulates that three consultants, one of whom must be the responsible consultant, could sign the DNR if all treatment options have been explored and further treatment and CPR is likely to be futile. The law does not permit advance directives from the patient, and CPR should still be done if it is the wish of the patient or their family.

Historically, it has been reported that the knowledge of the outcomes of CPR among physicians is poor, and physicians may not accurately predict the likely outcome of CPR [12, 13]. A more recent study found that the practice of CPR and DNAR among physicians is influenced by inadequate knowledge of patient preference, time pressure and personal bias [14]. Scoring systems have been developed to mitigate this subjective physician assessment. A number of previously developed scoring systems have been shown to have poor sensitivity and specificity in predicting the outcome of CPR. In a Swedish validation study, both the PAM (Pre-arrest morbidity) and the PAR (prognosis after resuscitation) scores had suboptimal performance in predicting outcomes in IHCA with AUC of 0.6 and 0.71, respectively [15]. Another validation study from the United Kingdom compared three morbidity scores and found a 20–29% sensitivity in predicting unsuccessful CPR [16].

Using data from the Get With The Guideline-Resuscitation (GWTG-R), Ebell and colleagues developed an objective scoring system that can predict the probability of survival with good neurological outcomes in cardiac arrest patients with attempted resuscitation [17]. The GO-FAR study was based on 51,240 adult patients from 366 American hospitals submitted to the GWTG-R registry. The study population were divided into three sets: the training, testing and the validation sets. The objective was to determine parameters obtainable at admission that could predict good neurological outcomes in patients following CPR in the case of IHCA. The study came up with 13 variables which constitute the GO-FAR variables. Each variable is given a weighted score ranging from − 15 to + 11. The total scores are grouped into four survival categories. The lower the sum score for a patient, the higher their probability of survival. (Table 1) A web-based app (https://www.mdcalc.com/calc/10033/go-far-good-outcome-following-attempted-resuscitation-score) can be used to calculate the score for any individual patient. The GO-FAR scoring system enables clinicians to objectively discuss possible outcomes of CPR with patients and families in the event of IHCA, thus allowing for more objective shared decision-making. The GO-FAR score has been externally validated and has shown consistency in differentiating patients with functional good outcomes following CPR in IHCA [17–19]. Thus, it can be considered the gold standard.

Functional outcome was determined using the cerebral performance category (CPC) score, which is a five-point scale ranging from 1 (able to work and live independently with minor physical or psychological disability) to 5 (brain death). A good neurological outcome was defined as a cerebral performance score of ≤ 2.

This study aims to compare three consultants’ DNR decisions with the GO-FAR score predictions of the probability of survival with good neurological outcomes following IHCA.

## Methods

### Study design and population

This is a retrospective study of all non-trauma adult patients, 18 years or older, placed on a DNR order (ICD-10 code Z66) signed by three board-certified consultants over 12 months during hospital admission from June 2021 to May 2022.

### Definition of terms

In our setting, the consultants in emergency medicine, the intensive care unit, and a third speciality specific to the patient’s presentation signed the DNR order in agreement with the patient or their family. We defined consultants as doctors who have completed an accredited residency program in a specific speciality in North America, Australasia and Western Europe. The patient’s clinical data and the GO-FAR variables were admission values. The patient’s comorbidity loads were the number of active chronic diseases that the patient had at the time of admission to the hospital.

In-hospital cardiac arrest are patients who suffered a cardiac arrest during their hospital admission and were given CPR. We defined Locals as Emirati nationals and Expatriates as non-Emirati nationals.

### Study setting

Tawam Hospital is a tertiary institution that serves a population of 750,000 in the Al Ain Region of Abu Dhabi Emirate in the UAE. It has 450 in-patient beds and serves as the regional cancer centre.

### Studied variables

Patients’ socio-demographics, admission physiologic parameters and the GO-FAR variables [Table 1.**]** were abstracted from the electronic medical records by members of the research team.

### Ethical considerations

The study was approved by the Tawam Human Research Ethics Committee (T-HREC, Ref No: KD/AJ/853). The patients or their caregivers gave their written informed consent to use their data for research.

### Statistical analysis

Data are presented as a proportion for categorical variables, median and inter-quarter range (IQR) for continuous variables. Fisher’s Exact test and Mann-Whitney U test were used where appropriate. We divided the study population into two groups: those with a GO-FAR probability of survival of ≤ 3%, which indicates futility and those with a > 3% probability of survival. We employed a logistic regression analysis to evaluate the predictive factors for those classified as > 3%, which was our dependent variable. The independent variables were the nationality of the patients, sepsis and oncology.

A P-value of < 0.05 was accepted as significant. Analysis was done using Statistical Package for Social Sciences (IBM, SPSS version 28, Chicago, IL).

## Results

During the study period, 788 patients received a DNR order from a group of three board-certified physicians. Of these, 392 patients (49.4%) were female. The patients’ median age (25th to 75th percentiles) was 71 (55 to 82) years. A majority of the patients were expatriates (*n* = 468, 59.4%). Most patients (*n* = 571, 72.5%) had no previous DNR order at admission. Table 1. Shows the GO-FAR scores and the probability of survival based on the total sum of the scores.

**Table 1:** GO-FAR score, sum score, and probability of survival categories

Variable	GO-FAR Score
Neurologically intact or with minimal deficits at admission	-15
Major trauma	10
Acute stroke	8
Metastatic or hematologic cancer	7
Septicemia	7
Medical noncardiac diagnosis	7
Hepatic insufficiency	6
Admit from a skilled nursing facility	6
Hypotension or hypoperfusion	5
Renal insufficiency or dialysis	4
Respiratory insufficiency	4
Pneumonia	1
Age (year)	
70–74	2
75–79	5
80–84	6
≥ 85	11
All groups combined	
Very low (< 1%)	≥ 24
Low (1–3%)	14 to 23
Average (> 3–15%)	-5 to 13
Above average (> 15%)	-15 to -6

GO-FAR, Good Outcome Following Attempted Resuscitation scores and the probability of survival [Ebell 17]

The GO-FAR probability of survival with good neurological outcome model categorized 230 (29.2%) of the patients as very low, 211 (26.8%) as low, 340 (43.1%) as average and 7 (0.9%) as above average. The demographics of the patients, categorized by the GO-FAR predictions of survival, are presented in Table 2.

**Table 2:** Patient demographics

	GO-FAR Probability of Survival				
	Very Low N (%)	Low N (%)	Average N (%)	Above Average N (%)	P-Value
Sex					0.435
Female	121 (52.6)	99 (46.9)	167 (49.1)	5 (71.4)	
Male	109 (47.4)	112 (53.1)	173 (50.9)	2 (28.6)	
Age	82 (67–89)	72 (57–82)	65 (49–76)	60 (57–64)	< 0.001
Nationality					< 0.001
Local	123 (53.5)	85 (40.3)	111 (32.6)	1 (14.3)	
Expatriates	107 (46.5)	126 (59.7)	229 (67.4)	6 (85.7)	
*Previous DNR					0.002
No	147 (63.9)	155 (73.5)	262 (77.1)	7 (100)	
Yes	83 (36.1)	56 (26.5)	75 (22.1)	0 (0.0)	
*Long term Care					< 0.001
No	200 (87.0)	156 (73.9)	300 (89.1)	7 (100)	
Yes	30 (13.0)	55 (26.1)	37 (10.9)	0 (0.0)	
No of patients (%)	230 (29.2)	211 (26.8)	340 (43.1)	7 (0.9)	

*N = 785 due to missing data

Patients’ median (25–75 percentile) systolic blood pressure, pulse, respiratory rate, SpO2, and GCS were 113 (100–130), 94 (79–110), 20 (18–24), 98 (96–100), and 15 (11–15), respectively. The patients’ median (25–75) percentile comorbidity load was 2 (1–2), and the length of stay was 11 (6–21) days.

Table 3 shows the clinical parameters of patients categorized by GO-FAR predictions. As expected, there were significant differences in the median (IQR) of the respiratory rate, oxygen saturation and Glasgow Coma Scale (GCS) of the very low/low categories compared with the average/Above Average categories. There were 219 (27.9%) oncology patients, of which 148 (67.6%) were classified by GO-FAR as having an average or above-average probability of survival with a good neurological outcome. Significantly more patients in the average and above average probability of survival group died compared with those with very low and low probability 243 (70%) versus 249 (56.5%) (*P* < 0.001)).

**Table 3:** Clinical information of patients

	GOFAR Probability of Survival				
	Very Low N (%)	Low N (%)	Average N (%)	Above Average N (%)	P-Value
SBP	112.5 (95-133.8)	112 (98.5-128.5)	114 (103–128)	121 (105.5–136)	0.378
Pulse	93.5 (77–111)	91 (76.5-109.5)	95 (82–110)	98 (93–110)	0.399
RR	22 (20–26)	20 (18–26)	20 (18–22)	22 (21–23)	< 0.001
SpO2	97 (94–99)	98 (96–99)	99 (97–100)	100 (99.5–100)	< 0.001
GCS	11 (9–15)	13.5 (9–15)	15 (15–15)	15 (14.5–15)	< 0.001
Comorbidity Load	2 (1–3)	2 (1–2)	1 (1–2)	1 (1–2)	0.06
*Primary Diagnosis					-
CKD	2 (0.9)	0 (0.0)	2 (0.6)	0 (0.0)	
Cardiovascular	5 (2.2)	0 (0.0)	4 (1.2)	2 (28.6)	
Oncology	27 (11.7)	44 (20.9)	145 (42.6)	3 (42.9)	
Neuro/Stroke	10 (4.3)	16 (7.6)	12 (3.5)	0 (0.0)	
Respiratory	13 (5.7)	13 (6.2)	11 (3.2)	0 (0.0)	
Liver Disease	0 (0.0)	2 (0.9)	6 (1.8)	0 (0.0)	
DM	0 (0.0)	0 (0.0)	1 (0.3)	0 (0.0)	
HIV/TB	0 (0.0)	1 (0.5)	3 (0.9)	0 (0.0)	
Sepsis	160 (69.6)	125 (59.2)	139 (40.9)	2 (28.6)	
Others	13 (5.7)	10 (4.7)	14 (4.1)	0 (0.0)	
Length of Stay	10 (5–17)	10 (6–19)	14 (7–25)	8 (6.5–13.5)	< 0.001
Outcome					< 0.001
Died	127 (55.2)	122 (57.8)	239 (70.3)	4 (57.1)	
Discharged	103 (44.8)	89 (42.2)	101 (29.7)	3 (42.9)	

Table 2: Numbers are presented in percentages, median (IQR) as appropriate. *N = 785 due to missing data

Table 4 shows patient demographics and clinical information comparisons of patients with very low and low GO-FAR probability of survival and patients with average and above average GO-FAR probability of survival. Overall, 441 (56%) of the patients had very low and low survival with good neurological outcomes, while 347 (44%) were classified as having an average or above average survival prediction according to the GO-FAR model. Three consultants placed these 347 patients on a DNR order, indicating futility. Still, the GO-FAR score classified them as having an average or above-average probability of survival with a good neurological outcome. The patients so classified were more likely to be expatriates, oncology patients, and non-sepsis (*P* < 0.001).

**Table 4:** Patient demographics and clinical information according to probability of survival by GO-FAR

	GO-FAR Probability of Survival		
	Very Low and Low N (%)	Average and Above Average N (%)	P-Value
Sex			0.943
Female	220 (49.9)	172 (49.6)	
Male	221 (50.1)	175 (50.4)	
Age			
Nationality			< 0.001
Local	208 (47.2)	112 (32.3)	
Expatriates	233 (52.8)	235 (67.7)	
*Previous DNR			< 0.001
No	302 (68.5)	269 (77.5)	
Yes	139 (31.5)	75 (21.6)	
*Long term Care			< 0.001
No	307 (89.2)	356 (80.7)	
Yes	37 (10.8)	85 (19.3)	
Oncology (Total)			< 0.001
No	335 (76.0)	102 (29.4)	
Yes	106 (24.0)	245 (70.6)	
Sepsis (*n* = 785)			< 0.001
No	104 (23.6)	255 (73.5)	
Yes	337 (76.4)	89 (25.6)	
Outcome			< 0.001
Died	249 (56.5)	243 (70.0)	
Discharged	192 (43.5)	104 (30.0)	

*N = 785 due to missing data

**Fig. 2:**
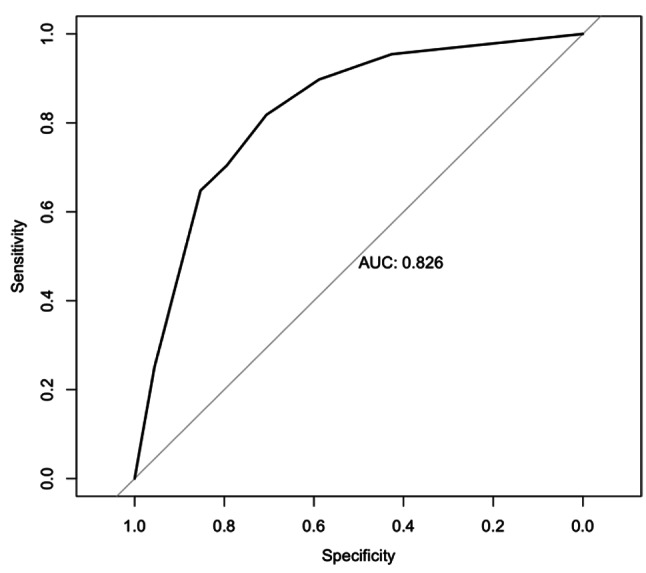
Oncology, Nationality and Not sepsis ROC curve for average and above average probability of survival

Figure 2. Shows the logistic regression analysis of the variables that predicted average and above-average GO-FAR probability of survival in the study group. For oncology patients (Estimate = -0.236, Std. Error = 0.029, z = -8.193, *p* < 0.001), the negative coefficient suggests that oncology treatment is associated with lower odds of appropriate classification. For sepsis patients (Estimate = 0.260, Std. Error = 0.029, z = 8.978, *p* < 0.001), a positive coefficient indicates that sepsis is associated with higher odds of appropriate classification. For nationality (Estimate = -0.657, Std. Error = 0.206, z = -3.187, *p* = 0.001), the negative coefficient implies that expatriate status is associated with lower odds of appropriate classification. The area under the curve (AUC) value was 0.826, indicating strong predictive power. The confusion matrix shows satisfactory performance, having 54 true negatives, 26 false positives, 14 false negatives, and 62 true positives.

## Discussion

Our results showed the three-consultant DNR model correctly classified 56% of the study population as having futility following CPR. Being expatriates, oncology patients, and those without sepsis increases the likelihood of being ‘misclassified’ by the three-consultant model as having futility prospects despite having good neurological outcomes based on the GO-FAR score.

Jones et al. reported that regardless of experience and speciality, physicians were generally poor at estimating survival rates for both IHCA and out-of-hospital cardiac arrest (OHCA) patients [12]. However, their study was in a general population, and the physicians had no clinical information. Using clinical vignettes of IHCA patients with known outcomes, Ebell et al. reported that physicians showed poor accuracy, reliability, and discrimination when predicting the probability of survival in IHCA patients [13]. The participants were from family medicine and internal medicine; they did not have access to the complete patient’s medical records and made the decisions independently. Our study differs from these previous studies in that the three consultants had full access to the electronic medical records, and their decision was a consensus. Despite these advantages, only 56% of the patients with DNR were predicted to have a poor outcome by the GO-FAR score. The remaining 44% had a probability of survival of > 3%.

Our study shows a positive association between DNR due to futility, being an expatriate, being an oncology patients, and not having sepsis. The AUC of 0.826 in the logistic regression model shows these variables are effective in accurately classifying patients with DNR into the average and above average probability of survival group. The odds ratio of mortality for patients with malignancy undergoing CPR was reported as 13.86 [15]. In the GO-FAR study, solid or haematological metastatic cancer was given a score of 7, which was the 4th highest behind age > 85 years, major trauma, and acute stroke. Since all the patients in the derivative study had CPR, patients with poor prognoses, such as those with metastatic cancer, may have been excluded due to the practice of DNR and advance directives. In this study, there were 219 (27.9%) oncology patients, of whom 148 (67.6%) were in the average and above-average prognosis groups. This indicates that oncology patients are associated with the physicians’ DNR orders.

A total of 426 patients had sepsis, of whom 337 (79.1%) were in the very low and low survival categories by the GO-FAR score, indicating an agreement between the GO-FAR score and the three consultant classifications. Since sepsis is treatable, having a score of 7 in the GO-FAR, it is likely that those patients had other significant comorbidities, hence their overall high GO-FAR scores.

The UAE population is unique, with 90% of the general population being expatriates and young migrant workers who tend to retire to their home countries. However, there are proportionately more Emiratis in the older strata of the population [20]. Of those classified as having average or above average probability of survival with good neurological outcomes, 112 (32.3%) were Emiratis, while 235 (67.7%) were expatriates. In the GO-FAR analysis, 65% of the Emiratis were identified as having a low or very low chance of survival with a good neurological outcome, whereas only 49.8% of expatriate patients were so classified, indicating that more expatriates with an average or above average probability of survival by the GO-FAR scores were considered as having futility according to the three-consultant model. The higher proportion of elderly patients among the local Emiratis is likely to have given them higher GO-FAR scores compared with the expatriates [20]. Furthermore, racial differences in DNAR rates have been reported, with the DNAR rates being highest among white Americans compared with African Americans, Asians and Hispanics. [21] Physician’s knowledge and local culture may also be contributing factors. [22]. Racial differences and local culture may have influenced our results, which could be the focus of future research.

The DNR has evolved since its introduction into medical practice in the mid-1970s [23]. However, the practice in our setting is still developing, and we do not have advance directives like other countries. It is important that clinicians are able to provide patients and their families with reliable information about the likelihood of success in the event of cardiac arrest, thus enabling them to make decisions about CPR. The GO-FAR system has been externally validated as a useful tool in different settings. A validated GO-FAR score will be relevant in our setting as an alternative objective method for determining patients who may not benefit from CPR. However, physicians may need to consider broader social and patient-specific issues when placing a DNR code. Some of these include advance directive policies, the prognosis of the underlying disease and the local laws. In such a context, the three-consultant model could help determine the futility of CPR and the need for a DNR order.

## Limitations

We acknowledge that there were some limitations to our study. First, there were no follow-ups of the patient’s post-hospital discharge, so functional status post-discharge is unknown. Second, the timing of the DNR from admission was not analyzed separately, and some patients may have been relatively well with stable vital signs but later deteriorated, warranting a DNR later. This may have given them a low GO-FAR score initially. Furthermore, many patients were receiving home nursing care in our setting and may not have been captured by the GO-FAR score. This may have underestimated the effect of long-term nursing facilities on their GO-FAR scores.

## Conclusions

There was a good correlation between the three-consultant categorization and the GO-FAR score for patients with sepsis. However, three consultants placed a significant number of oncology and expatriate patients with a good probability of survival on GO-FAR scores on DNR orders. The GO-FAR score guides joint decision-making on the possible outcomes of CPR in the event of IHCA. The physicians’ recommendation and the ultimate patient’s resuscitation choice may differ due to more complex contextual medico-social factors.

## Data Availability

No datasets were generated or analysed during the current study.

## Abbreviations

AUC: Area Under the Curve
CPR: Cardiopulmonary Resuscitation
DNR: Do Not Resuscitate
GCS: Glasgow Coma Scale
GO-FAR: Good Outcome Following an Attempt to Resuscitate
GWTGR: Get With the Guideline Resuscitation
IHCA: In-Hospital Cardiac Arrest
UAE: United Arab Emirates
